# Parent-Offspring Associations in Body Composition: Findings From the Southampton Women's Survey Prospective Cohort Study

**DOI:** 10.1210/clinem/dgad128

**Published:** 2023-03-21

**Authors:** Rebecca J Moon, Stefania D’Angelo, Christopher R Holroyd, Sarah R Crozier, Keith M Godfrey, Justin H Davies, Cyrus Cooper, Nicholas C Harvey

**Affiliations:** MRC Lifecourse Epidemiology Centre, University of Southampton, Southampton SO16 6YD, UK; Paediatric Endocrinology, University Hospital Southampton NHS Foundation Trust, Southampton SO16 6YD, UK; MRC Lifecourse Epidemiology Centre, University of Southampton, Southampton SO16 6YD, UK; Department of Rheumatology, University Hospital Southampton NHS Foundation Trust, Southampton SO16 6YD, UK; MRC Lifecourse Epidemiology Centre, University of Southampton, Southampton SO16 6YD, UK; NIHR Applied Research Collaboration Wessex, Southampton Science Park, Innovation Centre, Southampton, SO16 7NP, UK; MRC Lifecourse Epidemiology Centre, University of Southampton, Southampton SO16 6YD, UK; Faculty of Medicine, University of Southampton, Southampton SO16 6YD, UK; NIHR Southampton Biomedical Research Centre, University of Southampton and University Hospital Southampton NHS Foundation Trust, Southampton SO16 6YD, UK; Paediatric Endocrinology, University Hospital Southampton NHS Foundation Trust, Southampton SO16 6YD, UK; Faculty of Medicine, University of Southampton, Southampton SO16 6YD, UK; MRC Lifecourse Epidemiology Centre, University of Southampton, Southampton SO16 6YD, UK; NIHR Southampton Biomedical Research Centre, University of Southampton and University Hospital Southampton NHS Foundation Trust, Southampton SO16 6YD, UK; NIHR Biomedical Research Centre, University of Oxford, Oxford OX4 2PG, UK; MRC Lifecourse Epidemiology Centre, University of Southampton, Southampton SO16 6YD, UK; NIHR Southampton Biomedical Research Centre, University of Southampton and University Hospital Southampton NHS Foundation Trust, Southampton SO16 6YD, UK

**Keywords:** adiposity, obesity, developmental programming

## Abstract

**Context:**

Children born to parents who are overweight or obese have a high risk of adult obesity, but it is unclear if transgenerational associations relating to unfavorable body composition differ by parent.

**Objective:**

To examine differential mother-offspring and father-offspring associations in body composition in early childhood.

**Methods:**

A total of 240 mother-father-offspring trios from a prospective UK population-based pre-birth cohort (Southampton Women's Survey) were included for anthropometry and dual-energy x-ray absorptiometry assessment of whole-body-less-head body composition in the offspring at 3 different ages (4, 6-7, and 8-9 years) and in the mother and father at the 8- to 9-year offspring visit. Associations were assessed using linear regression adjusting for the other parent.

**Results:**

Positive associations between mother-daughter body mass index (BMI) and fat mass were observed at ages 6 to 7 (BMI: β = .29 SD/SD, 95% CI = .10, .48; fat mass β = .27 SD/SD, 95% CI = .05, .48) and 8 to 9 years (BMI: β = .33 SD/SD, 95% CI = .13, .54; fat mass β = .31 SD/SD, 95% CI = .12, .49), with similar associations at age 4 years but bounding the 95% CI. The mother-son, father-son, and father-daughter associations for BMI and fat mass were weaker at each of the ages studied.

**Conclusion:**

A strong association between the fat mass of mothers and their daughters but not their sons was observed. In contrast, father-offspring body composition associations were not evident. The dimorphic parent-offspring effects suggest particular attention should be given to early prevention of unfavorable body composition in girls born to mothers with excess adiposity.

Obesity and unfavorable body composition are major public health concerns in both adults and children. Children who are overweight or obese are at high risk of remaining overweight/obese in adulthood (1) and of developing the associated long-term chronic complications, including type 2 diabetes, cardiovascular disease, malignancy, and osteoarthritis.

Obesity is caused by a complex interplay of genetic, developmental, and environmental factors. Many studies have suggested a role for the intrauterine environment in the programming of future body composition, including associations between maternal dietary, lifestyle, and body composition factors in pregnancy and offspring body mass index (BMI) and body composition (2-5). Indeed, strong relationships between maternal and offspring BMI have been reported (6, 7), perhaps reflecting shared genetics, unfavorable intrauterine conditions for infants of obese women, or shared postnatal lifestyle traits. There are fewer data reporting relationships between paternal body composition and that of their offspring (7, 8), and furthermore many of these studies, like those reporting maternal-offspring associations, have relied on BMI as an indicator of adiposity. BMI is an important outcome due to its association with long-term morbidity and mortality (9), but it provides no information on the relative proportion of fat mass and lean mass, which can vary considerably for a given value (10). There are also considerable inconsistencies in study findings, which might reflect the different methods employed and variable age at body composition assessment (11-14). Furthermore, most studies have assessed the relationships at a single cross-sectional time point, which does not account for the changing body composition over early childhood and adolescence due to the adiposity rebound and pubertal development (15). We therefore explored the associations between parent and offspring body composition assessed by dual-energy x-ray absorptiometry (DXA). In our prospective cohort study, each child was invited for body composition assessment at 3 ages in early childhood: age 4 years (before the adiposity rebound for most children), at age 6 to 7 years (around or just after the adiposity rebound) (16) and 8 to 9 years (before or at the onset of puberty). Thus, we were able to explore cross-sectionally the relationships between parent (assessed only at offspring age 8-9 years) and offspring body composition at each time point and observe how these might differ at each stage of early childhood.

## Methods

### The Southampton Women's Survey

The Southampton Women's Survey (SWS) is a prospective preconception mother-offspring cohort study. Details of the study have been previously published (17). Briefly, 12 583 nonpregnant women aged 20 to 34 years living in the city of Southampton, UK were recruited into the study during 1998-2002; 3158 delivered a liveborn infant.

The SWS was conducted according to the guidelines laid down in the Declaration of Helsinki, and the Southampton and South-West Hampshire Research Ethics Committee approved all procedures (06/Q1702/104). Written informed consent was obtained from all participants and by a parent or guardian with parental responsibility on behalf of their children.

### Pregnancy and Birth Assessments

Detailed phenotyping of the women was undertaken in early (11 weeks gestation) and late pregnancy (34 weeks gestation) including an interviewer-administered questionnaire on health, lifestyle, physical activity, and diet with anthropometric measurements taken by a trained researcher.

### Offspring and Parent Follow-up

Subsets of children were invited to participate in assessments of body composition and bone health at 3 ages (age 4, 6-7, and 8-9 years). At only the 8- to 9-year follow-up, the child's mother and father were also invited to attend for a dual-energy x-ray absorptiometry (DXA) scan.

At each visit, height was measured with a Leicester height measurer (Seca Ltd, Birmingham, UK) to the nearest 0.1 cm with the head placed in the Frankfurt plane. Weight was measured in light clothing to the nearest 0.1 kg using electronic scales (Seca Ltd, Birmingham, UK). Body mass index was calculated as weight (kg)/height (meters)^2^. Standard deviation scores (SDS) for age and sex were calculated using the British 1990 reference data (18, 19).

A whole-body DXA scan was obtained using a Hologic Discovery Instrument (Hologic Inc., Bedford, MA, USA) to measure whole-body-less-head (WBLH) fat mass, lean mass, and bone mineral content (BMC) (20). As greater adiposity is associated with higher absolute lean mass, percent fat mass, and percent lean mass were subsequently derived using a 3-compartment model (fat mass, lean mass, and BMC) to provide an indication of a more or less favorable body composition. All scan images were reviewed by 2 researchers and any with excess movement or artifact were excluded. The DXA instrument underwent daily calibration using a spine phantom. The experimental coefficient of variation for this instrument when a spine phantom was repeatedly scanned in the same position 16 times, in a single session with no repositioning, was 0.68%.

### Statistical Analysis

Data were included in the analysis if mother, father, and offspring had all attended for the 8- to 9-year DXA assessment. All characteristics were visually checked for normality of distribution. T-tests, Mann-Whitney U-tests, and chi-squared tests were used to compare normally, nonnormally distributed, and categorical variables, respectively. Correlations were assessed using Pearson correlation coefficients and Spearman rank correlation coefficients for normally and nonnormally distributed variables, respectively.

Owing to recognized differences in body composition in boys and girls, analyses were stratified by offspring sex. Offspring body composition outcomes were adjusted for age at DXA using a linear regression technique. All variables were standardized using a Fisher-Yates transformation to a normally distributed variable with a mean of 0 and a SD of 1 (21). Multivariable linear regression was used to examine the parent-child relationships, thus yielding a standardized regression coefficient (SD per SD) to enable comparison of associations. The parent-child relationships were repeated comparing the body composition at each of the 3 ages of offspring assessment to the parent assessment at age 8 to 9 years. All models were adjusted for the equivalent exposure in the other parent. A directed acyclic graph (DAG) approach was used to select suitable confounders using DAGitty 3.0 (www.dagitty.net) (22, 23). Confounders were entered into the DAG informed by prior literature (3, 24-26), and those identified for inclusion in the models were maternal age, maternal dietary quality (measured as prudent diet score in late pregnancy and described in detailed previously (27)), maternal smoking in pregnancy, and offspring height (Supplementary Figure 1 (28)). Offspring height was excluded from models assessing the relationships with offspring BMI. The relationships were similar before and after inclusion of confounders; therefore, only the adjusted models are presented. The Wald test was used to compare the magnitude of the association between mother-offspring and father-offspring. All data were analyzed using Stata 16.0 (Statacorp, TX, USA).

## Results

### Characteristics of the Parents and Children

A total of 240 mother-father-offspring trios participated in this substudy. The included mothers were of similar age, parity, and early-pregnancy BMI compared with the mothers participating in the SWS who reported a live birth but who did not participate in this substudy. Those who participated were more highly educated and less likely to smoke in pregnancy than the women who did not participate in this substudy (Table 1). Sex and birthweight of the offspring were similar for those in this substudy to the whole cohort. A further 976 children attended the follow-up at 8-9 years but one or both parents did not participate in the DXA scan. Those children were similar in height, weight, and BMI SDS to the children who participated in this substudy.

**Table 1.** Characteristics of the mothers and offspring participating in this study and in comparison to the remainder of participants in the Southampton Women's Study mother-offspring cohort study

	Participants in this substudy		SWS participants not included in this substudy		
	n		n		*P*
**Age at delivery (years)**, mean (SD)	240	31.0 (3.5)	2916	30.6 (3.9)	.21
**Nulliparous prior to this birth**, n (%)	240	128 (53.3)	2915	1484 (50.9)	.47
**Prepregnancy BMI (kg/m^2^)**, median (IQR)	235	23.7 (21.8, 26.3)	2895	24.2 (21.9, 27.5)	.08
**Smoking in pregnancy**, n (%)	239	19 (7.9)	2751	466 (16.9)	<.001
**Education to degree level or higher**, n (%)	239	82 (34.3)	2910	611 (21.0)	<.001
**Black, Asian, or Minority ethnic group**, n (%)	240	7 (2.9)	2917	134 (4.6)	.96
**Offspring male sex**, n (%)	240	129 (53.8)	2913	1504 (51.6)	.53
**Offspring birthweight (g)**, mean (SD)	238	3431 (562)	2881	3429 (563)	.36

Age and height at the 8- to 9-year assessment were similar between boys and girls in this substudy, but the girls were heavier, had a higher BMI, and greater fat mass and percentage fat (Table 2). Of the 240 children, 206 and 203 had also attended the study visits at 4 years and 6-7 years, respectively. Anthropometric and body composition by sex at these ages are shown in Table 2.

**Table 2.** Characteristics of the children included in the study and comparison between boys and girls

	4 years		6-7 years		8-9 years	
	Boys	Girls	Boys	Girls	Boys	Girls
**N**	111	95	126	107	129	111
**Age (years)**, mean (SD)	4.11 (0.05)	4.12 (0.07)	6.73 (0.31)	6.75 (0.36)	9.19 (0.21)	9.16 (0.22)
**Height (cm)**, mean (SD)	103.8 (3.8)	104.4 (4.6)	119.8 (4.8)	120.4 (5.8)	135.1 (5.4)	136.3 (6.1)
**Height SDS**, mean (SD)	0.15 (0.94)	0.53 (1.13)*^b^*	−0.07 (0.87)	0.03 (1.02)	0.15 (0.91)	0.43 (1.01)*^b^*
**Weight (kg)**, median (IQR)	16.7 (15.4, 18.5)	17.4 (16.1, 18.7)	22.1 (20.3, 24.7)	23.0 (20.8, 25.2)	29.4 (26.4, 32.6)	30.8 (28.3, 35.1)*^d^*
**Weight SDS**, mean (SD)	0.03 (0.97)	0.37 (0.95)*^b^*	−0.01 (0.94)	0.15 (0.99)	0.05 (0.94)	0.30 (0.95)
**BMI (kg/m^2^)**, median (IQR)	15.7 (15.0, 16.4)	15.8 (15.2, 16.4)	15.5 (14.9, 16.4)	16.0 (15.0, 16.9)	16.0 (14.9, 17.5)	16.5 (15.3, 18.2)*^d^*
**BMI SDS**, mean (SD)	−0.08 (0.94)	0.13 (0.85)	0.03 (0.92)	0.18 (0.87)	−0.05 (1.02)	0.12 (1.04)
**Fat mass (kg)** *^a^*, median (IQR)	3.59 (3.19, 4.13)	4.35 (3.66, 5.40)*^d^*	4.11 (3.38, 5.11)	5.36 (4.29, 6.98)*^d^*	5.36 (3.94, 7.56)	7.49 (5.94, 10.30)*^d^*
**Lean mass (kg)** *^a^*, mean (SD)	9.93 (1.28)	9.58 (1.40)	14.36 (1.84)	13.67 (1.86)*^c^*	20.15 (2.64)	19.76 (2.77)
**Percent fat (%)** *^a^*, median (IQR)	26.2 (23.5, 28.9)	30.3 (26.3, 35.9)*^d^*	22.1 (18.7, 24.9)	26.9 (23.1, 32.8)*^d^*	19.9 (16.7, 26.2)	27.3 (23.1, 33.4)*^d^*
**Percent lean (%)** *^a^*, median (IQR)	71.2 (68.6, 73.9)	67.0 (61.8, 70.9)*^d^*	75.2 (72.3, 78.5)	70.1 (64.6, 73.8)*^d^*	77.0 (71.2, 80.3)	70.1 (64.3, 74.3)*^d^*

From whole-body-less-head DXA scans.

*P* < 0.05 compared to boys of the same age.

*P* < .01 compared to boys of the same age.

*P* < .001 compared to boys of the same age.

Maternal and paternal demographics and body composition are shown in Table 2. Correlations between the anthropometric and body composition measurements of the mothers and father ranged between r = 0.02-0.27 (Table 3). Neither maternal nor paternal body composition were associated with age.

**Table 3.** Characteristics of the parents, and correlations between maternal and paternal measurements

	Mothers	Fathers	Correlation between mothers and fathers
**Age (years)**, mean (SD)	40.8 (3.6)	43.6 (5.2)	0.52*^d^*
**Current smoker (%)**	9.0	11.6	
**White ethnicity (%)**	97.3	96.5	
**Height (cm)**, mean (SD)	164.9 (6.2)	176.7 (6.8)	0.21*^d^*
**Weight (kg)**, median (IQR)	68.2 (61.6, 79.9)	85.4 (76.5, 96.5)	0.15*^b^*
**BMI (kg/m^2^)**, median (IQR)	25.3 (22.8, 29.4)	27.3 (25.1, 30.4)	0.17*^c^*
**Fat mass (kg)** *^a^*, median (IQR)	23.9 (18.7, 31.8)	22.7 (17.7, 28.2)	0.25*^c^*
**Lean mass (kg)** *^a^*, mean (SD)	38.0 (5.9)	53.7 (7.2)	0.02
**Percent fat (%)** *^a^*, median (IQR)	38.6 (34.4, 44.1)	29.0 (25.3, 32.6)	0.27*^d^*
**Percent lean (%)** *^a^*, median (IQR)	58.5 (53.4, 62.7)	67.9 (64.4, 71.6)	0.26*^d^*

From whole-body-less-head DXA scans.

Correlation coefficient *P* < .05.

*P* < .01.

*P* < .001.

Maternal and paternal body composition data were not available at offspring ages 4 and 6-7 years, but stability of maternal BMI was suggested by a strong correlation in BMI prepregnancy and at the 8- to 9-year visit (r_s_ = 0.78, *P* < .001).

### Associations Between Offspring and Parental Anthropometry

**Figure 1.**
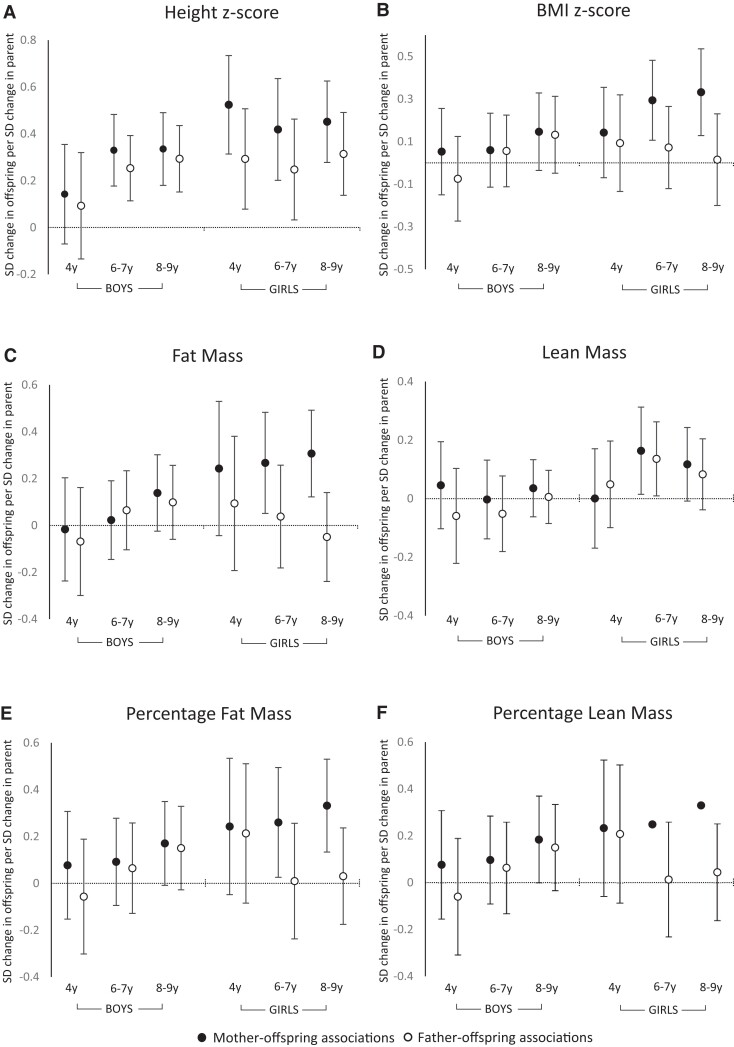
Associations between parent-offspring measures of anthropometry and body composition assessed by DXA, shown for boys and girls, with child assessments at ages 4, 6-7, and 8-9 years. All models are adjusted for the other parent and for maternal age, smoking in pregnancy, maternal dietary quality in late pregnancy, and offspring height (height not included in height z-score and BMI models).

Mother-offspring and father-offspring associations with height were observed in boys at ages 6-7 years and 8-9 years, and in girls at all 3 ages (Fig. 1A). For boys, the effect sizes observed for the associations of height with mother's and father's height were very similar. For girls the effect sizes tended to be of greater magnitude for mother-daughter than father-daughter, although the associations were not statistically different by Wald test (Fig. 1A).

Maternal BMI was positively associated with their daughter's BMI at age 6-7 years (β = .29 SD/SD, 95% CI = .11, .48) and 8-9 years (β = .33 SD/SD, 95% CI = .13, .54). A similar, but weaker, relationship was also observed at age 4 years (β = .14 SD/SD, 95% CI = −.07, .35). There were no significant father-daughter BMI associations (Fig. 1B). At age 8-9 years, the association between mother-daughter BMI was different from that of father-daughter BMI (*P* = .048, Wald test). In contrast, in the boys there were no associations between either maternal or paternal BMI and child's BMI at any of the ages studied (Fig. 1B).

### Associations Between Parental and Offspring Body Composition

Fat mass in girls at 8-9 years was associated with maternal (β = .31 SD/SD, 95% CI = .12, .49) but not paternal fat mass (β = −.05 SD/SD, 95% CI = −.24, .14). Similar associations were also evident for the mother-daughter associations with fat mass at ages 4 and 6-7 years, but there were no associations between the fat mass of the fathers and that of their daughters (Fig. 1C). In the boys, fat mass was not associated with either maternal or paternal fat mass at any of the ages studied (Fig. 1C). The associations with percentage fat mass were similar, with strong associations between female offspring and their mothers at ages 6-7 and 8-9 years (but weaker at age 4 years), but not with their fathers (Fig. 1E), and no associations between boys and their mothers or fathers.

The only body composition parameter for which there were associations between paternal and offspring measurements was lean mass, and this was only evident in girls at age 6-7 years (β = .14 SD/SD, 95% CI = .01, .26) with a weaker relationship at age 8-9 years (β = .09 SD/SD, 95% CI = −.04, .20) (Fig. 1D). Similar magnitude associations were observed for mother-daughter and father-daughter lean mass at ages 6-7 years age 8-9 years in girls (Fig. 1D), but with no clear associations for father-son or mother-son lean mass at any age (Fig. 1D).

## Discussion

In the Southampton Women's Survey prospective cohort study, we observed strong mothers-daughter associations for BMI and fat mass, with weaker associations for lean mass. Mother-son associations were not observed, and there were few father-offspring associations in body composition. We previously documented similar differential relationships in parent-child measures of bone mineral density and geometry in this cohort, with stronger mother-offspring correlations (29) and stronger associations in female than male offspring (30).

Several previous studies have shown associations between parent and offspring BMI (2-5) but there are few data reporting associations with specific measures of body composition, and these findings are inconsistent. Treuth et al similarly reported mother-daughter associations in fat mass and fat-free mass measured by DXA in prepubertal girls, but in contrast to our findings also reported father-daughter association in fat mass and fat-free mass of similar magnitude to the mother-daughter relationships (11). The girls included in that cohort were more ethnically diverse than in the SWS cohort, which is predominately of White ethnicity, which could account for the different findings. In contrast to our findings, Brener et al reported positive correlations between mother-son adiposity in prepubertal but not peripubertal boys, and no significant relationships for mother-daughter or father-daughter adiposity (14), but the interpretation of these findings is limited by the participants being selected based on follow-up in a pediatric endocrinology clinic for “growth observation” rather than a population cohort. Clifford et al reported relationships of similar magnitude for mother-offspring and father-offspring percent fat mass measured by bioelectrical impedance analysis at age 11-12 years (12). Similar to our findings, Sørensen et al found stronger associations of offspring BMI with maternal than paternal BMI (8). Interestingly, in a longitudinal study from age 4 years to 30 years, Casey et al observed mother-daughter relationships for waist to hip circumference at all ages, but father-son correlations in waist to hip circumference were not apparent until 2 years before peak height velocity (31). It is therefore possible that further follow-up of our cohort would reveal father-offspring relationships at older ages. It is also notable that the weight and weight SDS of the girls in this study was higher than that of the boys. It is possible that the difference in weight distribution in the 2 sexes confounded the relationships leading to apparent sexual dimorphism. Further replication of our findings in other large studies using DXA would be of benefit.

There are a number of factors that might determine parent-offspring correlations in body composition, including shared genetics and environmental influences, such as diet and physical activity. However, the stronger mother-offspring relative to father-offspring associations with BMI and adiposity also support a possible role for the intrauterine environment in moderating adipose development. Studies of siblings born before and after bariatric surgery have demonstrated a lower prevalence of obesity in those born after bariatric surgery (32), supporting the notion that the intrauterine environment is important for adipose tissue development, although it cannot be excluded that our findings and those of the studies before and after bariatric surgery are not due to shared mother-offspring postnatal environmental factors.

The relationships between mother-offspring BMI and fat mass were less apparent in boys than girls, but sexual dimorphism in fetal programming is recognized (33). This has been particularly described in animal models, but also in human studies (33). Adipose tissue composition and localization also differs by sex, and even in utero and at birth greater total fat mass in females is already apparent. Furthermore, females typically have a higher proportion of brown adipose tissue than males (34). These fundamental biological sex differences may contribute to the different mother-offspring relationships observed in sons and daughters if adipose tissue morphology and biology in males and females are differentially programmed by early life exposures, as has been suggested in some animal studies (35). Epigenetic modifications, including DNA methylation and histone modifications are key mechanisms believed to mediate the interaction between the in utero environment and clinical outcomes and may mediate the transgenerational inheritance of adipose tissue regulation and obesity. For example, the estrogen receptor is thought to be involved in the epigenetic regulation of adipogenesis (36), and sex differences in methylation have been observed in cord blood in relation to in utero environmental exposures (37, 38). Thus, these mechanisms could underlie the observed sex differences in mother-offspring body composition associations.

Shared postnatal environmental factors might also account for the different parent-offspring body composition relationships and between sexes. We did not assess which parent was the principal caregiver for the child nor did we determine whether one parent primarily determined their dietary intake. A survey in the United Kingdom in 2013, around the time these study data were collected, suggested that men with children were more likely to be working than women with children, and around 3 times more women worked less-than-full-time than men (39). Together, this suggests that mothers are more likely to spend more time in caregiving for their children than fathers, and thus may have more shared environmental factors for adiposity resulting in the differential relationships observed. Hesketh et al similarly reported significant relationships in accelerometer-derived physical activity in mothers and their children at ages 4 and 6 years (40, 41). Bergqvist-Noren et al identified significant associations between maternal and accelerometry-measured physical activity between the ages of 2 and 6 years, whereas there were no associations between father-offspring physical activity (42), and Jago et al showed stronger associations in moderate-vigorous physical activity between mother-daughters than mother-son, father-daughter, or father-son at age 5-6 years (43). Additionally, boys typically are more active than girls (40, 44), which may protect against fat mass gain accounting for the lack of mother-son correlation in body composition.

Our findings are supported by the work of Ornellas et al, who, using a mouse model, found that both male and female offspring of an obese mother/lean father mating were heavier at 2 and 12 weeks of age than offspring born to a lean mother/obese father mating. The obese mother/lean father offspring were similar in size to offspring born to both lean parents (45), suggesting, in similarity to our findings, that the maternal programming might exert greater effects than paternal programming. Ornellas et al additionally noted altered leptin signaling and hyperphagia associated with hypothalamic inflammation in the offspring of obese mothers. Interestingly, offspring of lean mother/obese father also had hypothalamic inflammation but without changes in intake or leptin signaling (45). In contrast, other work in rat and mouse models have suggested that paternal diet influences offspring glucose metabolism and body weight, often with greater effects in female offspring (46). We do not have measures of adipocytokines or glucose metabolism in our study cohort, but such measures could be included in future follow-up to attempt to elucidate mechanisms underpinning our observations.

The correlations between mother and daughter fat mass and percentage fat mass changed little between offspring ages of 4 years and 8 years, suggesting that these relationships are established early and before the typical period of the adiposity rebound, which usually occurs between 5 and 7 years (16). However, unraveling the cause and effect of any intergenerational effects of maternal obesity and offspring adiposity rebound on these relationships is complex. It would be important to establish persistence through puberty but nonetheless these findings are clinically important, highlighting girls who are born to mothers with high BMI and excess adiposity are at high risk of themselves becoming overweight/obese or having unfavorable body composition early in childhood. As the mother-daughter relationship in fat mass appears to be established by age 4 years, early awareness and intervention is needed in mothers with excess adiposity, and potentially beginning even in the periconception and in utero period.

The strength of this study are the detailed phenotyping of parents and their children at multiple time points in childhood and using the gold standard DXA for body composition assessment, although data were not available for the full study cohort. There are several limitations to this study. First, we did not formally assess paternity. In genetic screening studies in the United Kingdom, paternity discrepancy was identified in 1.4% to 1.6% of cases. We would therefore not expect this to impact our findings substantially (47), but if nonpaternity was higher than expected, this might explain the lack of associations in father-offspring. Second, the families included in the SWS are predominately of White ethnicity, reflecting the local population from which the participants were recruited. Care should be taken in extrapolating these findings to other ethnic groups. The mothers who were included in this substudy were also more highly educated and less likely to smoke during pregnancy than the mothers in SWS who did not participate in this substudy, which should be considered in the generalizability of the findings. Third, the children at the 8- to 9-year follow-up had a mean age of just over 9 years. It is considered normal for girls and boys to start puberty from 8 years and 9 years, respectively. Therefore, it is likely a small number of the participants were already in puberty, but this was not formally assessed. Fourth, parental body composition was assessed only at offspring age 8-9 years, and thus we have looked at these relationships cross-sectionally at multiple timepoints in childhood, rather than in a true longitudinal design with parental assessment at each age point. It is possible that parent body composition changed in the 4 to 5 years between the offspring DXA at age 4 years and that at age 8-9 years, although at population level body composition changes are small in early- to mid-adulthood (48), and in the mothers there was a strong correlation between BMI before pregnancy and at offspring age 8-9 years. Finally, in this cohort, we do not have measures of adipocytokines or assessment of parental or offspring glucose homeostasis. Measurement of biomarkers such as these in the future may provide clues to the mechanisms underpinning the observed relationships.

In conclusion, in this prospective birth cohort study, relationships were observed between mother and daughter body composition, but not with mother-son or father-offspring comparisons. Although the mechanisms are uncertain and require further exploration, these findings highlight that girls born to mothers with excess adiposity may be at higher risk of excess adiposity themselves, and approaches to addressing this at an early age should be considered.

## Data Availability

Some or all datasets generated during and/or analyzed during the current study are not publicly available but are available from the corresponding author on reasonable request.

## Abbreviations

BMC: bone mineral content
BMI: body mass index
DAG: directed acyclic graph
DXA: dual-energy x-ray absorptiometry
SWS: Southampton Women’s Survey
WBLH: whole-body-less-head
